# Evolution of signal multiplexing by 14-3-3-binding 2R-ohnologue protein families in the vertebrates

**DOI:** 10.1098/rsob.120103

**Published:** 2012-07

**Authors:** Michele Tinti, Catherine Johnson, Rachel Toth, David E. K. Ferrier, Carol MacKintosh

**Affiliations:** 1 MRC Protein Phosphorylation Unit, College of Life Sciences, James Black Centre, University of Dundee, Dow Street, Dundee DD1 5EH, UK; 2 Evolutionary Developmental Genomics Group, The Scottish Oceans Institute, University of St Andrews, East Sands, St Andrews KY16 8LB, UK

**Keywords:** *Branchiostoma*, *Ciona*, hereditary spastic paraplegia, RAB3GAP1, RAB3GAP2

## Abstract

14-3-3 proteins regulate cellular responses to stimuli by docking onto pairs of phosphorylated residues on target proteins. The present study shows that the human 14-3-3-binding phosphoproteome is highly enriched in 2R-ohnologues, which are proteins in families of two to four members that were generated by two rounds of whole genome duplication at the origin of the vertebrates. We identify 2R-ohnologue families whose members share a ‘lynchpin’, defined as a 14-3-3-binding phosphosite that is conserved across members of a given family, and aligns with a Ser/Thr residue in pro-orthologues from the invertebrate chordates. For example, the human receptor expression enhancing protein (REEP) 1–4 family has the commonest type of lynchpin motif in current datasets, with a phosphorylatable serine in the –2 position relative to the 14-3-3-binding phosphosite. In contrast, the second 14-3-3-binding sites of REEPs 1–4 differ and are phosphorylated by different kinases, and hence the REEPs display different affinities for 14-3-3 dimers. We suggest a conceptual model for intracellular regulation involving protein families whose evolution into signal multiplexing systems was facilitated by 14-3-3 dimer binding to lynchpins, which gave freedom for other regulatory sites to evolve. While increased signalling complexity was needed for vertebrate life, these systems also generate vulnerability to genetic disorders.

## 2. Introduction

Around 500 Ma, the vertebrates emerged from a massive evolutionary upheaval that involved two rounds of whole genome duplication (2R-WGD), with additional subsequent WGDs in certain lineages of bony fish and amphibians. Compelling evidence for these events emerged only recently, when the genomic signatures of the 2R-WGD were traced from invertebrates through to humans and other vertebrates [1,2]. A key new data source is the genome sequence of amphioxus (lancelet, *Branchiostoma*), the least-derived living invertebrate relative of the vertebrates within the phylum Chordata. Protein-coding gene duplicates that stem from the 2R-WGD are termed 2R-ohnologues. Generally, amphioxus has one ‘ancestral’ protein for each human 2R-ohnologue family. However, losses mean that only 15 to 30 per cent of genes in modern-day humans still belong to 2R-ohnologue families containing two to four members [1,2]. This raises several important questions: why did only certain gene duplicates survive? How did they shape vertebrate evolution? And what is their impact on human health and diseases?

Lists of human 2R-ohnologues were compiled recently and mapped onto datasets of genes that underpin biochemical events and diseases [2–4]. The human 2R-ohnologues were found to be less likely than non-ohnologues to have undergone subsequent small-scale duplications. This finding is consistent with the concept that present-day 2R-ohnologues have been maintained in dosage-balanced sets. Each of these sets is thought to contribute to a common process or structure that would be upset by changing the level of one or a few components [2]. Strikingly, many 2R-ohnologue families include Mendelian disease genes, which is also in line with the gene–dosage balance hypothesis [2,4,5]. Human 2R-ohnologues are also enriched in components of growth factor and developmental signalling pathways, and preferentially expressed in the nervous system and in vertebrate-specific organs [3]. The overall impression is that balanced sets of 2R-ohnologue families supported the evolution of vertebrate specialities, while also introducing vulnerability to genetic diseases.

In addition to the hypothesized retention of 2R-ohnologues owing to dosage-balance, it is thought that duplicate genes are often retained when they diverge to gain new functions (neofunctionalization) or partition subfunctions of their ancestral gene between the duplicates [6,7]. However, domain architectures are often conserved across 2R-ohnologue families, so it seems likely that functional genetic divergence may occur in the linker sequences between the domains, which tend to evolve faster than the functional domains and are enriched in regulatory phosphorylation sites [8].

Phosphorylated motifs are conserved to different degrees within protein families and across species. Some regulations require a precisely positioned phosphorylation, whereas in other cases the density of charge matters more than position [8–13]. Many phosphorylated residues dock onto regulatory proteins whose specificities may further constrain the evolution of the phosphoprotein.

The eukaryotic 14-3-3s comprise one such family of phosphoprotein-binding proteins. Their name refers to their discovery as proteins in fraction 14-3-3 in a sequential DEAE–cellulose and starch–gel separation of brain extract. 14-3-3s are dimers that dock onto specific pairs of phosphorylated serine and threonine residues on many proteins. These targets include human proteins that are linked to metabolic and neurological disorders, and to cancer [14]. By docking onto two phosphorylated residues that may be phosphorylated by different kinases, a 14-3-3 dimer can act as a logic gate that integrates two inputs. The bound 14-3-3 dimer may mask a functional domain in the target protein, or induce a conformational change [14–17]. Thus, a 14-3-3 dimer is a protein device that integrates two kinase signalling inputs and exerts a mechanical action on the target.

14-3-3-binding motifs generally have at least one basic residue in the −3 to −5 positions relative to the phosphorylated serine or threonine, and never a +1 proline [14,17]. Such sequences are phosphorylated by AGC (protein kinase A/protein kinase G/protein kinase C family) and CAMK (Ca^2+/^calmodulin-dependent protein kinase) protein kinases, including PKA (protein kinase A), Akt/PKB (protein kinase B), SGK (serum and glutocorticoid-regulated kinase), p90RSK (90 kDa ribosomal protein S6 kinase), PKCs (protein kinase C family members) and AMPK (AMP-activated protein kinase) [18]. Therefore, 14-3-3s mediate cellular responses to insulin, growth factors and other stimuli that activate these kinases [14,19].

Recent studies of two sister Rab-GTPase activating proteins (AS160/TBC1D4 and TBC1D1) that regulate glucose uptake into muscles inspired speculation that 14-3-3 dimers could provide an evolutionary mechanism for the regulatory divergence of their targets [14]. AS160 and TBC1D1 each contain two 14-3-3-binding sites: one site is similar in both proteins, but the second site differs between them. The 14-3-3-binding site common to each protein is an insulin-regulated Akt/PKB-phosphorylated site. The second site on AS160 is phosphorylated by Akt/PKB and p90RSK, whereas on TBC1D1 it is phosphorylated by kinases including AMPK, which is activated in energy-depleted cells [20–22]. It is therefore inferred that AS160 and TBC1D1 have complementary roles in regulating glucose homoeostasis in response to insulin and energy stress, respectively [23].

Accordingly, we proposed the lynchpin hypothesis. Suppose that an ancestral 14-3-3-binding protein were duplicated. Then, if one 14-3-3-binding site remained unchanged, it could provide a ‘lynchpin’ whose binding to a 14-3-3 dimer might provide sufficient intracellular control to permit the second 14-3-3-binding site to evolve into a consensus for phosphorylation by a different protein kinase. The result would be two proteins with the same function, but regulated by different signalling inputs [14].

Here, we wished to progress from an anecdotal to a more systematic analysis. Therefore, we classified human proteins for which 14-3-3-binding sites have been reported, and discovered that the majority are also 2R-ohnologues. Sequence alignments indicated interesting patterns of conservation and divergence of 14-3-3-binding sites across 2R-ohnologue families. We investigated these in the REEP protein family, which includes the Hereditary Spastic Paraplegia 31 protein REEP1. Our findings have implications for understanding the evolution of vertebrates, and also for certain neurodevelopmental disorders, metabolic diseases and cancer. Further, we suggest a conceptual model for considering intracellular regulation in terms of multiple-input multiple-output (MIMO) array systems.

## 3. Results

### 3.1. Most well-defined human 14-3-3-binding proteins are 2R-ohnologues

**Figure 1.**
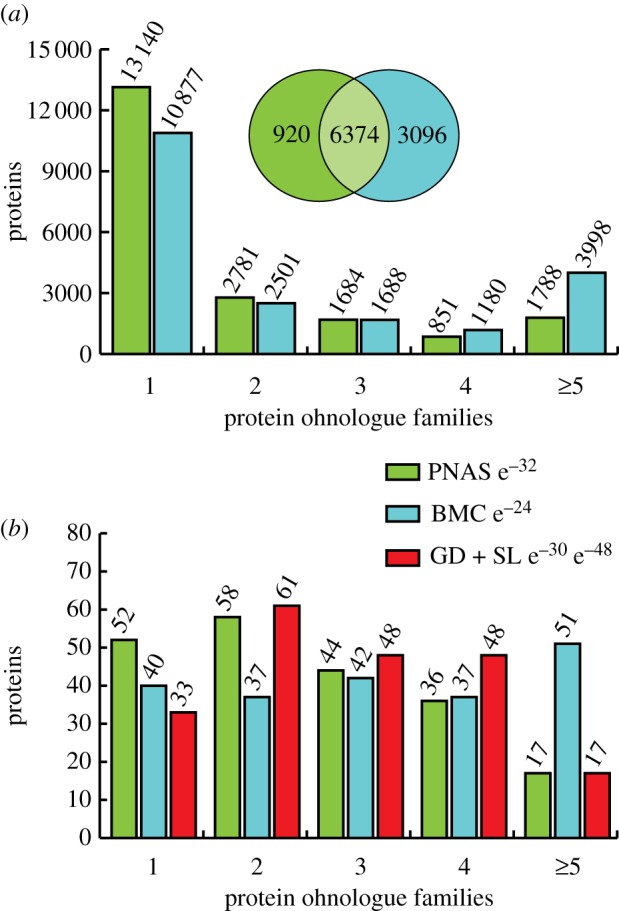
Enrichment of 2R-ohnologues among well-defined 14-3-3-binding phosphoproteins. (*a*) 2R-ohnologues in the human proteome. Two published 2R-ohnologue gene datasets (PNAS, green) [2] and (BMC, blue) [3] were transcribed onto the human proteome (20 244 proteins in UniProt release 08-2011), and plotted according to 2R-ohnologue protein family sizes assigned in [2] and [3] (see the electronic supplementary material, table S2). The Venn diagram depicts the overlap of 2R-ohnologues assigned by the two studies. (*b*) Gold and silver 14-3-3-binding protein 2R-ohnologue families. Up to August 2011, 172 gold standard (GD) proteins with defined 14-3-3-binding phosphosites had been identified, while a further 35 are silver standards (SL) whose direct phosphorylation-dependent binding to 14-3-3 has been demonstrated but the relevant sites not pinpointed [14,24] (electronic supplementary material, table S1). These 207 proteins were assigned into 2R-ohnologue families of 2, 3, 4 and 5 or more proteins according to the assignments in (*a*) (green and blue, as before). In red are the 2R-ohnologue families assigned in this study by abbreviated forms of the procedures given for the REEP proteins in figures 4 and 5. The hypergeometric probabilities of the observed distributions occurring by chance are indicated. For the manually curated data (this study), the two hypergeometric probabilities represent comparisons with the whole proteome datasets from [2] and [3], respectively.

Two published lists of human 2R-ohnologues overlap by 6374 genes [2,3], with a further 920 genes uniquely assigned as 2R-ohnologues in one study [2] and 3096 in the other [3] (figure 1*a*). Much of the difference is due to a larger number of families of five or more genes being counted as 2R-ohnologues in the latter study. By definition, a 2R-ohnologue family is not expected to contain more than four protein-coding genes, unless there have been further duplications after the 2R-WGD. Thus, when there are insufficient data to sort larger families of paralogues into their respective 2R-ohnologue subsets, these have been left unresolved as families of five or more as a temporary measure (figure 1*a*).

Genes from these two studies were matched to the corresponding human proteins with UniProt identifiers. In this way, 7104 [2] and 9367 [3] out of the 20 244 non-redundant human proteins could be assigned to 2R-ohnologue families of two or more proteins (figure 1*a*).

**Figure 2.**
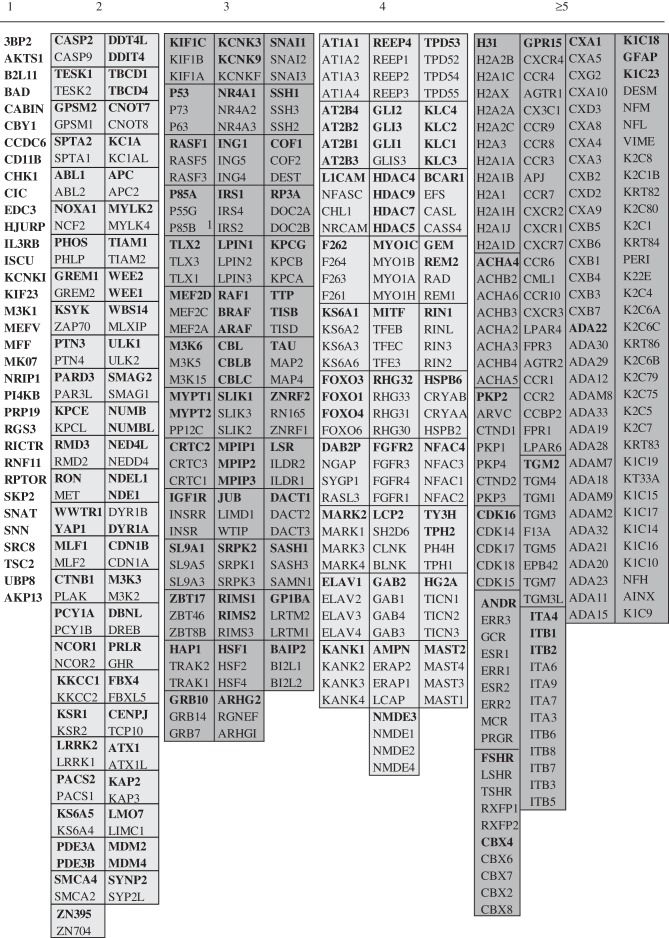
14-3-3-binding proteins in their 2R-ohnologue families. Each 2R-ohnologue family is in a box, with the gold and silver standard 14-3-3-binding proteins in bold, and family members that have not been shown to bind to 14-3-3 in regular text. Previous studies also concluded that the following comprise 2R-ohnologue sets: mdm2 and mdm4 [25]; beta and gamma catenin [26]; MITF, TFE3, TFEB and TFEC [27]; YAP1 and WWTR1 (TAZ) [28]; the ANNAT family [29]. Pseudogenes were not included, such as the human IRS3 pseudogene, which otherwise conforms to the gene synteny pattern of a 2R-ohnologue. Also, MFF, listed here as a non-ohnologue, is related to a human pseudogene (LOC392452). There were cases where post-2R-WGD gene rearrangements were evident, such as TESK1 and TESK2, though, in line with previous analyses [2], these were few.

There are 172 ‘gold standard’ human proteins with well-defined 14-3-3-binding sites, and a further 35 ‘silver standards’ for which direct phosphorylation-dependent binding to 14-3-3s has been demonstrated, but relevant phosphosites not yet pinpointed [14,24] (electronic supplementary material, table S1). We compared this list of 207 14-3-3-binding proteins with the published 2R-ohnologue lists [2,3], and also with a 2R-ohnologue list that we compiled by performing phylogenetic and gene synteny analyses for every 14-3-3-binding protein (examples of such analyses are given later). Strikingly, approximately 84 per cent (174/207) of gold and silver 14-3-3-binding proteins are 2R-ohnologues, belonging to 139 protein families with a total of 525 family members (figures 1*b* and 2; electronic supplementary material, table S2). Therefore, compared with human proteins in general (figure 1*a*), the 14-3-3-binding phosphoproteome is highly enriched in membership of 2R-ohnologue families (figures 1*b* and 2).

### 3.2. Using 2R-ohnologue datasets to help create a priority list for validation of high-throughput 14-3-3-capture experiments

**Figure 3.**
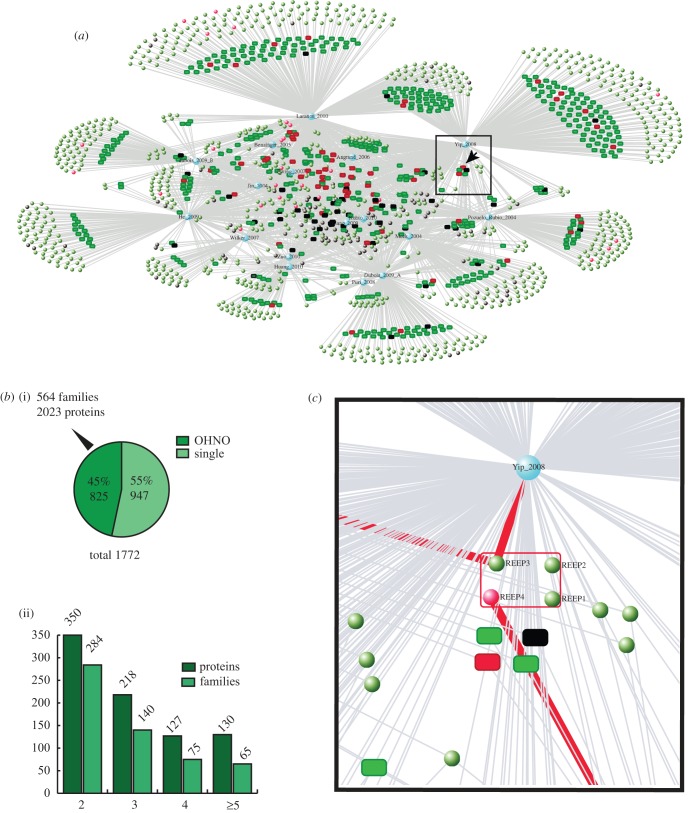
Visualization of the mapping of 2R-ohnologue families onto the 14-3-3 interactome. (*a*) A graph created using VisANT (visant.bu.edu) [30], from the data collated in electronic supplementary material, table S2, shows the overlapping sets of proteins that were isolated from mammalian cells in 16 HTP 14-3-3-affinity capture experiments published up to August 2010 [24]. Each study was assigned a node in blue and lines connect these articles to the identified proteins, depicted with a default of green circle nodes. Then, well-defined gold and silver standard 14-3-3-binding proteins (see the electronic supplementary material, table S1) were changed from green to red. Proteins from the ‘common contaminants’ list (electronic supplementary material, table S3) were changed to black. Thereafter, proteins were assigned to 2R-ohnologue families, which were depicted as rectangles whose internal structure is described in panel (*c*). The interactive version of this graph is available under 14-3-3 partners at http://www.ppu.mrc.ac.uk/research. (*b*) (i) The overlap between the HTP 14-3-3 affinity capture experiments (*a*) and 2R-ohnologues is 825 proteins according to the 2R-ohnologue assignment of [2]. (ii) Classification of these 825 2R-ohnologue proteins into families of 2, 3, 4 and 5 or more proteins. (*c*) The box in (*a*) was expanded to visualize the REEP protein family members, which reveals that REEP3 had been identified in two HTP 14-3-3-affinity capture experiments, while REEP4 had been reported in one HTP experiment and had also achieved gold standard status by identification of a 14-3-3-binding phosphosite. The interactive version of the VisANT graph (*a*) reveals similar details for any protein or protein family by clicking on nodes on the graph, or entering the required UniProt protein name into a search box.

In addition to gold and silver 14-3-3-binding proteins, 1772 proteins had been isolated from mammalian cell and tissue lysates by 14-3-3-affinity capture in 16 high-throughput (HTP) proteomics experiments up to August 2010 (figure 3*a*) [24]. It is therefore imperative to sort these proteins into a manageable priority list for the future determination of which are true 14-3-3-interactors; which bind indirectly to 14-3-3s as components of multi-subunit complexes; and which proteins had been isolated because they bind non-specifically to affinity matrices. The overlap between these HTP experiments and 2R-ohnologues is approximately 825 proteins, in 564 families with a total of 2023 members (figure 3*b*). Given the striking enrichment of 2R-ohnologues among the well-defined 14-3-3-binding proteins (figures 1*b* and 2), we suggest that the 2R-ohnologues from the HTP screens should receive priority attention.

As an additional way to filter potential non-specific hits from the HTP 14-3-3 proteomics datasets, we prepared an ‘exclusion list’ of potential contaminants using data provided by colleagues, who had isolated proteins from human cell lysates using ‘control’ beads or ligands unrelated to 14-3-3s. Interestingly, there is a relatively clean separation between the gold and silver 14-3-3-binding proteins, and these contaminants (see figure 3*a*; electronic supplementary material, table S3). Applying the 2R-ohnologues as an ‘inclusion list’ and the common contaminants as ‘exclusion list’ to the proteins identified in HTP 14-3-3 proteomics screens resulted in a list of 750 proteins that represent 538 families with a total of 1929 members (see electronic supplementary material, table S3).

In summary, while the numbers are impossible to predict precisely, there are strong indications that the total overlap between 2R-ohnologues and 14-3-3-binding phosphoproteins may be substantial, and it may be helpful to focus on this subset when selecting candidates for biochemical experiments.

### 3.3. Disease associations of 2R-ohnologue families that include 14-3-3-binding proteins

An association between 2R-ohnologues and human genetic disease has already been pointed out [2]. Here, we examined the disease associations of 2R-ohnologue families that contain the gold and silver 14-3-3-binding proteins, and found that a striking 91 per cent of these families (127 out of 139) and 64 per cent of the 2R-ohnologue proteins (350/525) are associated with a disease; developmental, cancer and psychiatric were the most represented disease categories (see electronic supplementary material, table S2 and figure S1). Interestingly, for 76 families, different members of the same family are linked with distinct disorders (see electronic supplementary material, table S2 and figure S1).

### 3.4. Evolutionary history of the human REEP proteins and their ‘lynchpin’ 14-3-3-binding site

To identify potential ‘lynchpins’, we determined how many published 14-3-3-binding phosphosites are conserved across the respective 2R-ohnologue family and align with serine or threonine residues in the corresponding pro-orthologues of the invertebrate chordates, namely *Branchiostoma* (amphioxus) and *Ciona* (tunicates). Not all sites could be assigned one way or another because many of the invertebrate protein sequences in the databases are incomplete. However, 103 experimentally defined sites in 76 protein families are conserved across the human protein family and find a matching serine or threonine in the sequences from the chordate invertebrates (see electronic supplementary material, table S2 and figure S2). One such conserved potential ‘lynchpin’ is in the receptor expression enhancing protein (REEP) family (figure 3*c*, and later).

The six REEP proteins include REEP1, which is mutated in hereditary spastic paraplegia 31 (one of a group of disorders characterized by progressive weakness and stiffness of the legs), and REEP4, loss of which causes paralysis in *Xenopus* [31,32]. REEP proteins are tethered to the cytoplasmic face of the endoplasmic reticulum (ER) and contiguous membranes, and are implicated in ER morphology (as are the other hereditary spastic paraplegia proteins atlastin and spastin), mitochondrial function, and translocation of olfactory, taste and other receptors to the cell surface [33,34].

**Figure 4.**
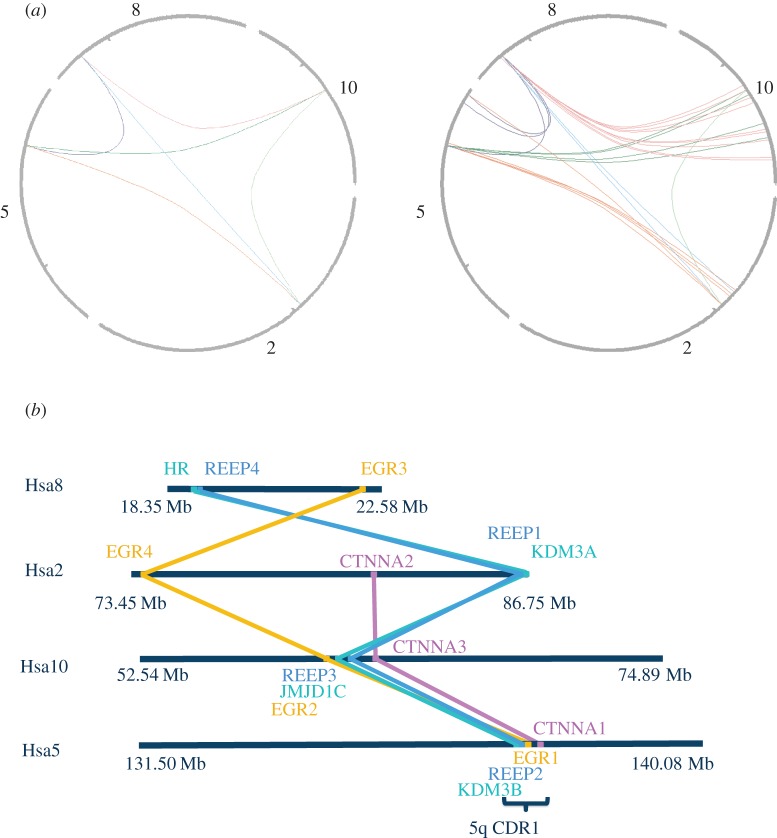
Gene synteny analysis of the vertebrate REEP 1–4 proteins. (*a*) Gene synteny clusters (paralogons) within the human genome were identified using only the human REEP1, REEP2, REEP3 and REEP4 genes as queries of the Synteny Database (http://teleost.cs.uoregon.edu/synteny_db/) [36] (left), with the search widened to include genes within 500 kb of these genes (right). (*b*) The diagram shows only those gene matches that can be traced across at least three paralogons. Though not to scale, chromosomal distances are indicated in megabases. The extent of the minimal 5q31.2 commonly deleted region 1 (CDR1) of myelodysplastic syndrome [35] is indicated. *Branchiostoma floridae* gene 185 339 is related to human REEPs 1–4; 269 996 is related to human EGR 1–3; and 124 949 to human HR, KDM3A, JMJD1C, KDM3B.

**Figure 5.**
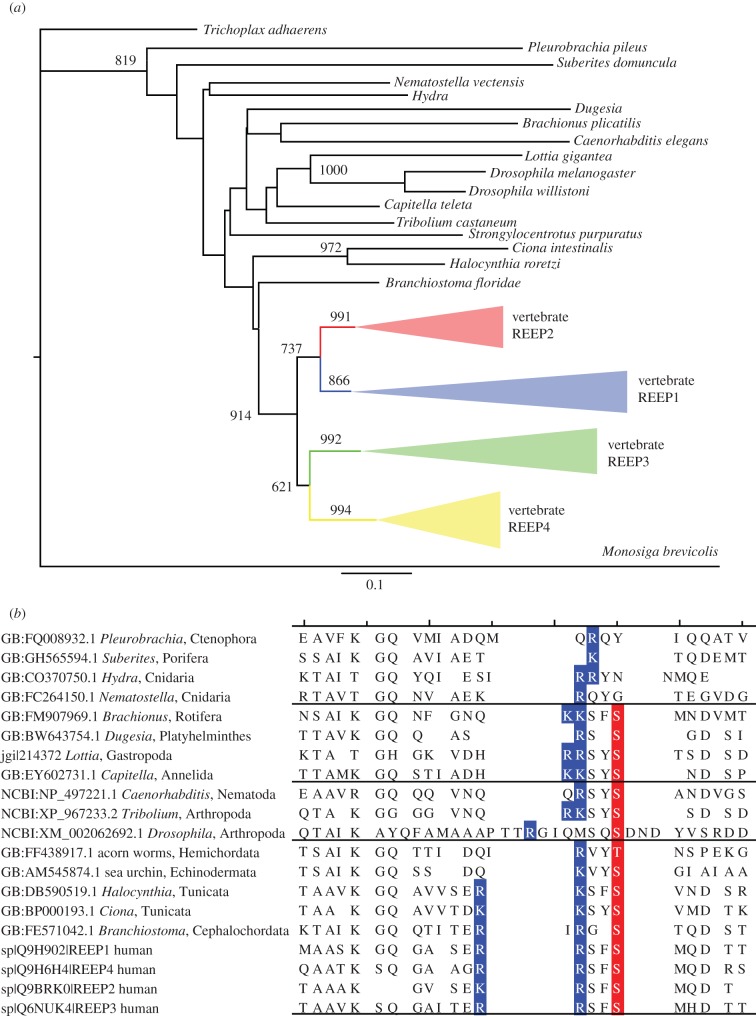
Phylogenetic tree of the vertebrate REEP 1–4 proteins and evolution of the lynchpin 14-3-3-binding phosphorylation site. (*a*) The phylogenetic tree of the vertebrate REEP 1–4 proteins shows the topology expected from the 2R-WGD events. Sequences were aligned using MAFFT within Jalview (http://www.jalview.org/). The neighbour-joining tree was constructed in PHYLIP (http://evolution.genetics.washington.edu/phylip.html), using the JTT model and 1000 bootstrap replicates. Numbers above the branches show the bootstrap support for that branch. Only values over 700 (i.e. 70%) are shown, except for the value uniting vertebrate REEP 3 and 4 groups, which falls just below this value (at 621). The tree is rooted with the non-metazoan choanoflagellate sequence from *Monosiga brevicolis* as the outgroup. The vertebrate REEP 1–4 proteins cluster with the four human proteins into four paralogy groups represented by triangles, with the expanded version given in electronic supplementary material, figure S3. (*b*) Sequence alignments with the ‘lynchpin’ 14-3-3-binding motif on human REEP 1–4 (Ser152 of REEP4). The lynchpin region of the human REEP 1–4 was aligned with corresponding sequences from selected metazoan species of the Bilateria, which comprise Deuterostomia (bottom nine sequences) and Protostomes (Ecdysozoa—three sequences shown; and Lophotrochozoa—four shown); and non-Bilaterian basal metazoans (top four sequences), according to the super-phylum classification outlined by Telford & Copley [37]. Serine and threonine residues that align with Ser152 of human REEP4 are in red, and basic residues that align with those in the 14-3-3-binding motif are in dark blue.

Phylogeny, gene synteny and sequence alignments indicate that two REEP genes in invertebrate chordates—185 339 and 57 028 (proteins C3ZZ30 and C3Z345) of *Branchiostoma floridae*—gave rise to two separate 2R-ohnologue families in vertebrates: REEPs 1–4 and REEPs 5–6, respectively. The latter, including the amphioxus pro-orthologue, lack the 14-3-3-binding region and are not considered further. Human REEPs 1–4 display the genetic signatures expected for a family derived from 2R-WGD and not from small-scale tandem duplications. The four genes are located on different chromosomes (*REEP1* is at 2p11.2, *REEP2* lies within the myelodysplastic syndrome (MDS) 5q31.2 microdeletion [35], *REEP3* is at 10q21.3 and *REEP4* at 8p21.3), and the genes for human REEPs 1–4 are in chromosomal segments that share conserved neighbouring genes: that is, they reside in ‘paralogons’ (figure 4). The phylogenetic tree shows single invertebrate REEP genes clustering as a sister group to the clade of all four vertebrate paralogy groups. The tree topology is consistent with that expected if the first round of whole genome duplication (1R-WGD) gave rise to two genes, and then in the 2R-WGD one of these genes gave rise to REEP1 and REEP2, whereas the other duplicated to make REEP3 and REEP4 (see figure 5*a*; electronic supplementary material, figure S3). Interestingly, REEP4 is missing from the bony fish.

Ser152 of REEP4 is a phosphorylated residue whose mutation to Ala prevents its binding to 14-3-3s in cell lysates, and which lies within a motif resembling a typical 14-3-3-binding site (RLRSF(s152)MQ, where the lower case ‘s’ is phosphorylated) [24]. All four human REEP proteins have a Ser that corresponds to Ser152, with minor variations at −5 (Lys in REEP2) and +2 (His in REEP3), and this residue is therefore a potential lynchpin (figure 5*b*). Variations on this 14-3-3-binding motif are found in the REEP orthologues of species within the Bilateria (figure 5*b*), and although phosphorylation cannot be inferred from sequence alone, this is consistent with the possibility that this 14-3-3-binding motif was in place in the chordates prior to the 2R-WGD at the origin of the vertebrates (figure 5*b*). All four vertebrate REEP proteins also have a –2 serine (Ser150 in REEP4) that is recorded as a phosphosite (www.phosphosite.org; figure 5*b*). Interestingly, the gold standard human 14-3-3-binding sites are enriched in phosphorylatable –2 serines, whereas other random sets of phosphorylated sites are not (see electronic supplementary material, figures S2 and S4).

### 3.5. Human REEP1, 2, 3 and 4 are differentially regulated by phosphorylation and 14-3-3s

**Figure 6.**
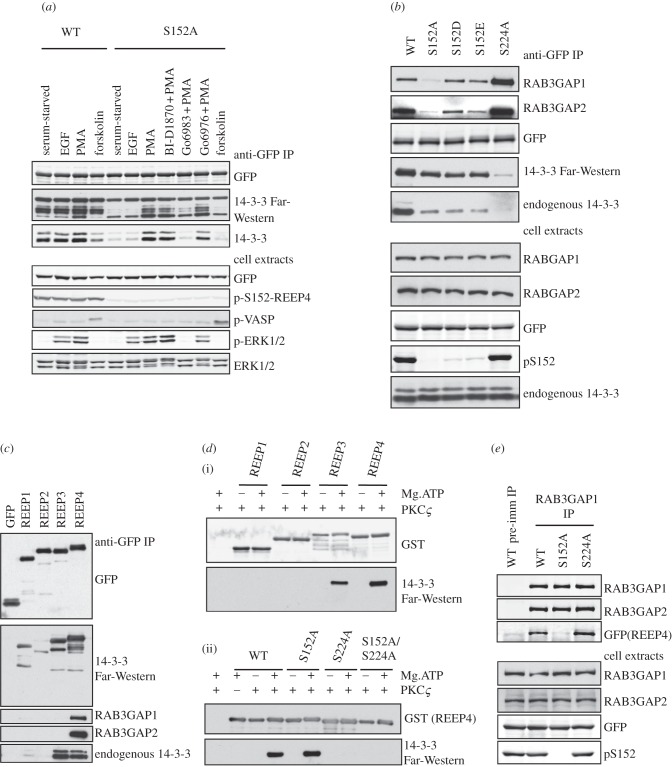
Differential regulation of vertebrate REEP 1–4 proteins. (*a*) Effect of different stimuli/inhibitor combinations on the cellular regulation of REEP4-GFP. HEK293 cells were transfected to express REEP4-GFP (wild-type) and REEP4-S152A-GFP, serum-starved for 8 h, then stimulated with or without inhibitors, as indicated. REEP4–GFP was isolated from cell lysates (anti-GFP IPs) and blotted with the antibodies indicated. Also, the ability of the GFP–REEP4 proteins to bind directly to 14-3-3 was tested with a 14-3-3 Far-Western assay. The blots of cell lysates (30 µg) show the phosphorylation of Ser152 of REEP4, and efficacy of EGF and PMA in stimulating Erk phosphorylation, and forskolin in stimulating VASP phosphorylation. (*b*) Effect of mutations on interactions of REEP4 with other proteins. Wild-type REEP4-GFP or the single site mutant proteins indicated were isolated from HEK293 cell lysates and blotted with the antibodies shown, and probed in a Far-Western assay for direct binding to 14-3-3s. (As here, it is typical that Asp and Glu do not mimic phosphorylated residues with respect to 14-3-3 binding.) (*c*) Interaction of REEP proteins with 14-3-3, RAB3GAP1 and RAB3GAP2. The indicated REEP-GFP proteins were isolated from lysates of transfected cells (anti-GFP IP). Copurification of endogenous RAB3GAP1, RAB3GAP2 and 14-3-3 was detected by immunoblotting, and the ability of REEP proteins to bind directly to 14-3-3 was also tested by Far-Western assay. (*d*) PKCζ phosphorylation and 14-3-3 binding of REEP1, 2, 3 and 4 *in vitro*. (i) GST-tagged REEP proteins (Y65-end, lacking the N-terminal membrane-binding regions) were expressed and purified from *Escherichia coli,* and phosphorylated *in vitro* with PKCζ. Protein (500 ng) was analysed by 14-3-3 Far-Western assay to detect direct binding to 14-3-3s. (ii) Wild-type REEP4 and putative 14-3-3-binding site mutants were assessed for PKCζ-mediated 14-3-3 binding. (*e*) Reciprocal interaction of RAB3GAP1 with REEP4. Endogenous RAB3GAP1 was immunoprecipitated from HEK293 cells transfected with plasmids to express wild-type or mutant forms of REEP4-GFP. The immunoprecipitates were blotted with the antibodies shown. A control immunoprecipitation was performed with pre-immune antibodies.

**Figure 7.**
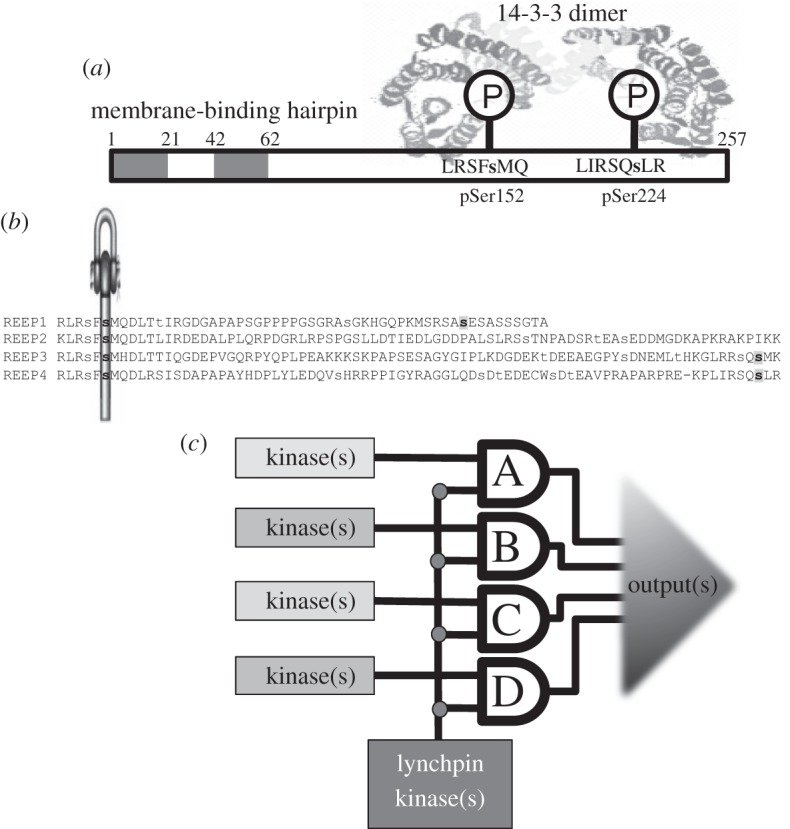
Signal multiplexing by 14-3-3-binding phosphoprotein families in the vertebrates. (*a*) The diagram depicts how a 14-3-3 dimer is envisaged to bind to REEP4 via phosphorylated Ser152, and also Ser224 that is phosphorylated in phorbol ester-stimulated cells. The N-terminal membrane-tethering hydrophobic hairpin is also indicated. (*b*) 14-3-3-binding sites of the human REEP proteins. Serine and threonine residues reported to be phosphorylated in the literature (www.phosphosite.org) and/or in this study are in lower case, and phosphorylated 14-3-3-binding residues are in bold: the lynchpin (phosphoSer152 in REEP4) is indicated by the symbol and second 14-3-3-binding sites in grey (pSer192 in REEP1, pSer225 in REEP3, pSer224 of REEP4). (*c*) A signal multiplexing system: this represents the scenario where a 2R-ohnologue family of 14-3-3-binding proteins (A to D) has evolved such that they each maintain a common lynchpin phosphorylation site for binding to 14-3-3 and have a variable second site, which can be phosphorylated by different kinases. Such multiplexers have considerable potential for regulatory variation: They could operate in multiple-input, multiple-output (MIMO) mode, or multiple-input, single-output (MISO) mode, generate graded output responses that increase in intensity as each target protein in the family is engaged, or be all-or-nothing devices that trigger an output only when every family member has been captured by a 14-3-3 dimer.

To understand how sequence relates to function, we examined the regulation of REEPs 1-4 biochemically. We raised a phospho-specific antibody, which revealed that phosphorylation of Ser152 of REEP4 was unaffected by various extracellular stimuli, including phorbol ester, and yet phorbol ester slightly increased the binding of 14-3-3s to REEP4 (figure 6*a*). Moreover, Ser152Ala-REEP4 mutant protein bound to 14-3-3 only in lysates of phorbol ester-stimulated cells, and this was blocked by the broad spectrum protein kinase C (PKC) inhibitor Gö6983, but not the PKCα and PKCβ-selective inhibitor Gö6976, nor p90RSK inhibitor BI-D1870 (figure 6*a*). These data suggested the existence of a second 14-3-3-binding site on REEP4, which was phosphorylated by a PKC, or PKC-activated protein kinase other than p90RSK, in response to phorbol ester. Mutation of Ser224 decreased overall 14-3-3 binding to REEP4 and prevented the phorbol ester stimulation of 14-3-3 binding (see figures 6*b* and 7; electronic supplementary material, figure S5). These data indicate that a 14-3-3 dimer binds to both phosphoSer152 and phorbol ester-regulated phosphoSer224 on REEP4 (figure 7*a*). Similar to REEP4, REEP3 also binds to 14-3-3 via phosphoSer152 and Ser225 (see figures 6*c* and 7*b*; electronic supplementary material, figure S5). Consistent with the cellular data, *in vitro* phosphorylation by PKCζ enabled REEP3 and REEP4, but not REEP1 and REEP2, to bind to 14-3-3 (figure 6*d*(i)). Ser224 is the PKCζ-phosphorylated site on REEP4 since its mutation to alanine prevented PKCζ-mediated 14-3-3 binding (figure 6*d*(ii)).

In contrast, the weak binding of 14-3-3 to REEP1 was not regulated by PMA (see figure 6*c*; electronic supplementary material, figure S5), but was enhanced by treating cells with the protein phosphatase inhibitor calyculin A (not shown). Analysis of point mutants indicated that phosphoSer192 of REEP1 is a second 14-3-3-binding site, which is phosphorylated by an unknown kinase (see electronic supplementary material, figure S5; figure 7*b*). REEP2 did not bind significantly to 14-3-3, though it is phosphorylated on a serine residue analogous to REEP4-Ser152 (see figure 6*c*; electronic supplementary material, figure S5), and no second site on REEP2 has yet been identified.

### 3.6. Human REEP4 binds to the RAB3GAP1–RAB3GAP2 heterodimer whose mutations underlie Warburg Micro and Martsolf syndromes

In addition to 14-3-3s, mass fingerprinting of Coomassie-stained gels and Western blotting of REEP-GFP proteins isolated from cell lysates showed that REEP4 binds to RAB3GAP1 and RAB3GAP2 (figure 6*b*,*c*), which together form a heterodimeric GTPase-activating protein for the small GTPase Rab3. Mutations in RAB3GAP1 and RAB3GAP2 cause the neurodevelopmental Warburg Micro (OMIM 60018) and Martsolf (OMIM 21270) syndromes, respectively [38–40].

Intriguingly, RAB3GAP1 and RAB3GAP2 do not bind to Ser152Ala-REEP4 (figure 6*b*,*e*). However, despite sharing Ser152 as a common binding determinant, the interactions of 14-3-3s and RAB3GAP1–RAB3GAP2 heterodimer with REEP4 differ. In contrast to 14-3-3s, RAB3GAP1 and RAB3GAP2 bind to unphosphorylated REEP4 (see electronic supplementary material, figure S5) and binding of RAB3GAP1 and RAB3GAP2 to REEP4 was not decreased when acidic residues took the place of Ser152 (figure 6*b*). Also, Ser224Ala-substituted REEP4 had enhanced binding to RAB3GAP1–RAB3GAP2, but almost no 14-3-3 interaction (figure 6*b*,*e*). Thus, 14-3-3s and the RAB3GAP1–RAB3GAP2 heterodimer must be in distinct REEP4-containing complexes.

Further analyses with a series of chimeric proteins that contain different combinations of parts of REEP3 and REEP4 indicated that the mid-region of REEP4 binds to RAB3GAP1 and RAB3GAP2, whereas the C-terminal third of REEP4 appears to be slightly inhibitory for RAB3GAP1–RAB3GAP2 binding (see electronic supplementary material, figure S5).

## 4. Discussion

### 4.1. The lynchpin hypothesis and evolution of signal multiplexing by 2R-ohnologue families

Our data suggest a new conceptual model for intracellular regulation in terms of protein families that evolved into signal multiplexing systems in the vertebrates. We propose that the binding of 14-3-3 dimers to one phosphorylated ‘lynchpin’ provided freedom for other regulatory sites to change and be regulated by different kinases on different family members [24,41]. Even minor sequence changes have the potential to rewire the signalling inputs from protein kinases. For example, PKB/Akt and SGK create phosphorylated RxRxx(pS/pT) motifs, whereas p90RSK prefers to phosphorylate the serine within RxRxx(pS), and Lats kinases phosphorylate HxRxx(pS/pT) sites [42,43]. The resulting 2R-ohnologue protein families would enable a given process to be regulated by more signalling inputs than could be accommodated by a single protein (figure 7*c*). Such signal multiplexing is reminiscent of the powerful multiple-input, multiple-output (MIMO) signalling arrays that boost signal-to-noise ratios and communication flows in smartphone networks.

Human 14-3-3-binding 2R-ohnologues include components of developmental and growth factor signalling pathways such as insulin-receptor substrate proteins, fibroblast growth factor receptors, Raf kinases and other protein kinase families [3], as well as many downstream target proteins that link these signalling pathways to the regulation of membrane trafficking, metabolism, the cell cycle and gene expression [44,45] (figure 2). The concept of MIMO arrays should therefore be a useful framework for understanding complexities of growth factor signalling pathways, including signal integration, feedback regulation, oncogene addiction and differential responses of different cell types. These ideas provide impetus to explore how the extra signal-streaming capacity of such MIMOs contributed to the evolution of complex vertebrate forms.

A special feature of vertebrate embryo development is that cells in the neural crest undergo an epithelial–mesenchymal transition (EMT) and migrate to different regions of the embryo, where they differentiate into a wide variety of cell types that contribute to craniofacial structures, heart valves, pigmented skin cells and many other vertebrate features. A reactivation of the EMT programme is thought to underlie the aggressive metastatic behaviours of cancers, such as melanoma. It is therefore interesting that key transcription factors that mediate the EMT and neural crest cell differentiation belong to 14-3-3-binding 2R-ohnologue families, including snail, MITF and TLX-2 [46–48].

We therefore suggest that neofunctionalization, in terms of divergence of regulatory phosphorylations and 14-3-3-binding specificities, made a greater contribution to 2R-ohnologue survival than has been realized. However, we do not reject paradigms of 2R-ohnologue retention driven by dosage balance and subfunctionalization. Indeed, the human 14-3-3s are themselves also 2R-ohnologues, which suggest that 14-3-3s coevolved with their targets, in line with the gene–dosage balance hypothesis. The concept of gene–dosage was developed for smaller pathways and complexes, and understanding how it applies to the human 14-3-3-interactome will require the emergent properties of the whole network to be defined. Efforts in this direction are under way. For example, proteomics experiments are identifying which proteins are phosphorylated and captured by 14-3-3s in response to different extracellular stimuli [44,45].

The singleton human 14-3-3 targets also went through the WGD events before being returned to singleton status by gene-loss mutations in all but one of the paralogues. For the species to survive, 14-3-3 signalling had still to be viable throughout these changes in gene–dosage, but perhaps these proteins were more efficient as singletons. It may be significant that several 14-3-3-binding singletons belong to ancient multi-protein complexes: Rictor, raptor and PRAS40/AKTS1 are in the nutrient-sensing mTOR (mammalian target of rapamycin) complexes; BAD and B2L11 are central components of the apoptotic machinery; and EDC3 (enhancer of mRNA-decapping protein 3) is part of an mRNA-decapping complex [44,45]. Also, 14-3-3-binding sites in two singletons are ancient, one in the human transcriptional repressor capicúa is conserved in the *Drosophila* protein [49], and the mammalian (PI4KIIIβ) and *Saccharomyces cerevisiae* (PIk1) forms of the Golgi regulator phosphatidylinositol 4-kinase also share a common 14-3-3-binding site [14,50].

We note that plants, which have their own history of whole-genome duplications, also have multiple 14-3-3s [51] and 14-3-3-binding proteins belonging to multi-protein families [52,53]. It would be interesting to discover whether there are mechanistic parallels between the evolution of vertebrate and plant 14-3-3-interactomes. *Saccharomyces cerevisiae* has also been through a WGD [54], raising the question of how this event influenced the evolution of its two 14-3-3 proteins and their phosphoprotein targets.

### 4.2. Case study of a disease-linked 14-3-3-binding 2R-ohnologue family: the REEP proteins

Here, 103 candidate lynchpins were identified, defined as 14-3-3-binding sites that are conserved across members of a human 2R-ohnologue family, and which align with Ser/Thr residues in the single pro-orthologues in the basal invertebrates of our chordate lineage (electronic supplementary material, figure S4). However, while sequence alignments are useful, only the type of biochemical analyses performed here for the REEP proteins can define 14-3-3-binding phosphosites with confidence.

Interestingly, 46 per cent (100/216) of experimentally defined 14-3-3-binding phosphosites and 47 per cent (49/103) of the potential 14-3-3-binding lynchpins have a phosphorylatable serine in the –2 position (see electronic supplementary material, figure S2 and table S2) [14,55], including that in the REEP 1–4 family (figure 7*b*). The REEP lynchpin (phosphoSer152 in REEP4) evolved in a step-wise manner with the basic residues being in place first, then a potential 14-3-3-binding site was formed before the 2R-WGD event, thereby making phosphoSer152 a potential lynchpin. The –2 serine (phosphoSer150 in REEP4) is in some, but not all, bilaterian invertebrate REEP proteins. This feature will require further work to define the relevant kinases and regulatory significance of the juxtaposed phosphoSer residues.

As well as acting as a lynchpin 14-3-3-binding site, the Ser152 region of REEP4 is involved in interactions with the RAB3GAP1–RAB3GAP2 heterodimer, which is a GTPase-activating protein for Rab3 that mediates neurotransmitter release. However, REEP4 cannot bind to 14-3-3 and to RAB3GAP1–RAB3GAP2 at the same time. Mutation of REEP1 causes hereditary spastic paraplegia 31 in humans, which is characterized by progressive weakness and stiffness of the legs, and mutation of REEP4 causes lower body paralysis in an animal model [31,32,56]. Mutations in RAB3GAP1 and RAB3GAP2 cause the autosomal recessive Warburg Micro and Martsolf syndromes, which involve congenital cataracts, microphthalmia, postnatal microcephaly and developmental delay [38–40]. It is intriguing to find a molecular connection between these two types of disorder. Regulated membrane trafficking is a molecular theme. One hypothesis is that REEP4 and related proteins are involved in trafficking of synaptic vesicles towards the ends of axons, during which events the REEP4 undergoes a phosphorylation-dependent switch that determines whether it binds to 14-3-3s or engages with RAB3GAP1–RAB3GAP2 to modulate Rab3-mediated neurotransmitter release. The motor neurons that run from the brainstem to connect with the lower motor neurons would be vulnerable to long-distance trafficking problems (consistent with the REEP mutations causing paraplegia), whereas many neurons would be affected by neurotransmitter release problems (consistent with Warburg Micro and Martsolf syndromes). It is unclear how the motor function of REEP proteins relates to the separate literature on how REEPs regulate cell surface expression of odourant and taste receptors [34], though mechanisms controlling sensory inputs and vertebrate motion may be evolutionarily linked [57].

The *KDM3B* (*JMJD1B*), *REEP2, EGR1* and *CTNNA1* genes lie within the MDS 5q31.2 CDR1 microdeletion region that is linked to the aggressive form of MDS and acute myeloid leukaemia [35,58–60], and this region also constitutes a 2R-WGD paralogon (figure 4). Within the conceptual framework of MIMO systems, it is interesting that peripheral neuropathy is a phenotype shared by hereditary spastic paraplegia 31 (REEP1) and MDS with 5q31.2 deletion (REEP2). These considerations suggest a case for examining whether REEP2 contributes to the aetiology of MDS.

### 4.3. The MIMO imbalance disease hypothesis

The 2R-ohnologues were previously reported to be enriched in disease genes [2–4]. Indeed, the 14-3-3s are themselves linked to diseases: chromosome deletions including the 14-3-3 epsilon gene result in craniofacial dysmorphisms, brainstem reduction in 14-3-3 protein levels has been identified in sudden infant death syndrome, and certain cancers involve changes in expression of specific 14-3-3s. Also, many 14-3-3-binding 2R-ohnologues are linked to disease, including cancer (the protein kinase B-Raf, E3 ubiquitin ligase mdm2, tumour suppressor p53/TP53 and transcription factor MITF), metabolic disorders (AS160, TBC1D1), movement disorders (ataxin-1, REEP1, protein kinase LRRK2) and developmental RASopathies (disorders linked to the Ras pathway; C-Raf and E3 ubiquitin ligase Cbl) [61–63].

It makes sense to suppose that if a critical regulatory function were performed by a single protein in an animal like amphioxus, then its mutation might be lethal. In contrast, mutation or loss of one or other component of a delicately balanced MIMO system might not be lethal if other proteins of the family can at least partially compensate for the function (figure 7*c*). However, the loss of a regulatory input may make the function become uncoordinated with other cellular processes, thereby causing the debilitating disorder.

While it was previously stated that disease is generally linked with just one member of any given 2R-ohnologue family [4], the situation appears to be more complicated, with mutations in different protein family members giving rise to different diseases, as has been discussed here for the REEP family. Another multi-disorder family is the Raf kinases, comprising B-Raf, A-Raf and C-Raf/Raf-1. B-Raf is mutated to an active V600E form in approximately 50 per cent of melanomas, and drugs that inhibit B-Raf-V600E have clinical efficacy [64]. However, when the patient also has cells with activated Ras, the drug-inhibited B-Raf interacts with other Raf proteins, facilitating their activation via Ras, which may promote tumour growth [65,66]. The activation of C-Raf/Raf-1 by Ras can be inhibited by 14-3-3 binding to protein kinase A-phosphorylated sites on C-Raf/Raf-1 [67]. Moreover, mutations that impair 14-3-3 binding to C-Raf, and hence promote its activation, cause developmental RASopathies (including the pSer259 14-3-3-binding phosphosite and Ser257, which we note is a phosphorylatable –2 serine residue) [68,69].

The REEP and Raf examples illustrate how the increased complexity of MIMO-based systems has gone hand-in-hand with increased vulnerability to disease during embryogenesis and adulthood, and emphasize that the study of ‘single gene’ diseases should take account of the potential interplay between the disease protein and other members of its 2R-ohnologue family.

Finally, over the evolutionary time-scale, variations in the signalling configurations of MIMO systems could also help explain the variety of species of mammals, reptiles, birds, amphibians and fish. For example, we were interested to notice that REEP4 is missing from fish, while IRS3 (insulin receptor substrate 3) is a pseudogene in humans, but is a functional gene in mice. In other words, genetic disorders and genetic variety are on the same continuum. It will be fascinating to compare the patterns of 2R-ohnologue loss and retention in different vertebrates, and try to relate these patterns to the special features of each lineage.

## 5. Material and methods

### 5.1. Gold and silver 14-3-3-binding proteins

The list of ‘gold standard’ mammalian proteins for which 14-3-3-binding sites were reported up to August 2010 [14,24] was revised and updated to August 2011. A list of ‘silver standards’ was also prepared from the literature, meaning proteins demonstrated to display direct and phosphorylation-dependent binding to 14-3-3s, but where phosphorylated residues were not identified. Relevant references are cited in electronic supplementary material, table S1.

### 5.2. Protein mapping

The datasets from Makino & McLysaght [2] and Huminiecki & Heldin [3] were used to define the ohnologue families. Both papers used Ensembl identifiers. The Biomart tool at Ensembl (http://www.ensembl.org/biomart/martview/) was used to create a table with Ensembl Gene ID (Ensembl Gene 65, *Homo sapiens* genes GRCh37.p5) and UniProt IDs (UniProt/SwissProt ID, UniProt/SwissProt Accession). The table was implemented as a dictionary in a python script to map the Ensembl identifiers onto UniProt IDs. The dataset of Huminiecki & Heldin [3] (see electronic supplementary material, table S1) clusters ohnologue families using the TreeFam ID as identifier, and no further steps were required. The ohnologue pairs from electronic supplementary material, table S1, worksheet 7 of Makino & McLysaght [2] were clustered into families and the family size computed with a python script. A progressive number was used as family identifier. A second python script was used to create a VisANT XML file to group each ‘node’ protein of electronic supplementary material, table S3 into the correspondent ohnologue family ‘group’.

### 5.3. Genetic association of diseases

The gold and silver standard proteins were assigned to the relevant diseases based on data in electronic supplementary material, tables S4a (for human) and S5a (for mouse) of Zhang *et al*. [70] (also see [28]). The gene2accession table from NCBI (ftp://ftp.ncbi.nlm.nih.gov/gene/DATA/) was used to create a python dictionary to convert the gene name to the Gene Identifiers and UniProt IDs. The BioMart service at Ensembl was used to retrieve the human homologues of the mouse UniProt accession IDs (two proteins were defined as homologues if they share 80% amino acid identity). The OMIM diseases were retrieved using the DAVID resource (http://david.abcc.ncifcrf.gov/). The disease classes (manually assigned) are based on Zhang *et al*. [70].

### 5.4. Biochemical analyses

Biochemical analyses of REEP-GFP proteins used the methods reported in Johnson *et al*. [24]. HEK293 cells cultured in medium containing 10 per cent (v/v) foetal bovine serum were transfected with plasmids to express REEP4-GFP (wild-type) and REEP4-S152A-GFP, as indicated. After 24 h, cells were serum-starved for 8 h, then stimulated with epidermal growth factor (EGF, 100 ng ml^–1^ for 15 min), phorbol-12-myristate-13-acetate (PMA, 100 ng ml^−1^ for 30 min) and adenylate cyclase activator forskolin (20 µM for 30 min), as indicated. Where shown, cells were incubated with p90 ribosomal S6 kinase (p90RSK) inhibitor BI-D1870 (10 µM for 30 min), pan-PKC inhibitor (Gö6983, 1 µM for 30 min), Gö6976 (which inhibits PKCα and PKCβ1, but not PKCδ, -*ɛ* or -ζ; 1 µM for 30 min) and non-specific protein kinase A inhibitor H-89 (30 µM for 30 min) prior to stimulation. REEP4-GFP was isolated from cell lysates (2.5 mg) using GFP-Trap agarose, and analysed. Antibodies that specifically recognize human REEP4 phosphorylated on Ser152 were raised in sheep against the synthetic phosphopeptide AGRLRSF(s)MQDLRC (residues 145–157, where lower case ‘s’ denotes phosphoSer152, plus Cys for coupling: ref. S500B, second bleed used).

## Supplementary Material

Tinti Suppl figs and legends

## Supplementary Material

Tinti TABLE_S1.xlsx

## Supplementary Material

Tinti TABLE_S2.xlsx

## Supplementary Material

Tinti TABLE_S3.xlsx

## Supplementary Material

Tinti TABLE_S4.xlsx
