# Supplementary material for: Evolution of signal multiplexing by 14-3-3-binding 2R-ohnologue protein families in the vertebrates
Source: Open Biol. 2012 Jul;2(7):120103. doi: 10.1098/rsob.120103 (PMC3411107; doi:10.1098/rsob.120103)
Supplement: Tinti Suppl figs and legends [file rsob120103-s1.docx]

**Tinti et al (2012) Supplementary Information: Table of content**

| Page 2 | Supplementary Figure S1  Disease and phenotypic associations of 2R-ohnolog families that include gold and silver standard 14-3-3-binding proteins |
| --- | --- |
| Page 3 | Supplementary Figure S2  Alignment of human 14-3-3 binding sites showing enrichment of phosphorylated residues in the -2 position relative to the phosphorylated 14-3-3-binding site |
| Page 4 and page 5 | Supplementary Figure S3  The extended phylogenetic tree of the vertebrate REEP1–4 and invertebrate REEP proteins |
| Page 6 | Supplementary Figure S4  Post-translational modifications surrounding the human 14-3-3 binding sites |
| Page 7 | Supplementary Figure S5  Biochemical regulation of human REEP proteins |
| Page 8 | Legends for Supplementary Tables 1 to 4, which are given separately as the following Excel documents:  Supplementary Table 1 The gold and silver standard human 14-3-3 binding proteins from the literature  Supplementary Table 2 The high throughput (HTP) 14-3-3-affinity capture studies and ohnolog families  Supplementary Table 3 Ohnolog families containing one or more gold and silver standard human 14-3-3-binding proteins, and their disease associations  Supplementary Table 4 The associated diseases and phenotypes of the 14-3-3 ohnolog families |

**Supplementary Figure S1 Disease and phenotypic associations of 2R-ohnolog families that include gold and silver standard 14-3-3-binding proteins**

Human and mouse genetic diseases and phenotypes were mapped onto the list of human proteins that are members of protein families that include gold and silver standard 14-3-3-binding proteins. Gold and silver 14-3-3-binding proteins are in red, and others in green. Experimental evidence was manually curated in addition to importing disease links from OMIM and human genetic association data and mouse genetic phenotypic information from [Zhang Y, De S, Garner JR, Smith K, Wang SA, Becker KG. (2010) Systematic analysis,comparison, and integration of disease based human genetic association data and

mouse genetic phenotypic information. BMC Med Genomics. 3,1].

Diseases (blue nodes) were classified computationally into Developmental, Cancer, Immune, Circulatory, Metabolic, Reproductive, Neurological, Psychiatric and Other disorders, as reported in Supplementary Tables S3 and S4. The REEP protein family box is expanded, showing REEP1 linked to Neurological (HSP31) (see text), and the JMJD1C-REEP3 on chromosome 10 is a quantitative trait locus for plasma levels of alkaline phosphatase [Yuan X, Waterworth D, Perry JR, Lim N, Song K, Chambers JC, Zhang W, Vollenweider P, Stirnadel H, Johnson T, Bergmann S, Beckmann ND, Li Y, Ferrucci L, Melzer D, Hernandez D, Singleton A, Scott J, Elliott P, Waeber G, Cardon L, Frayling TM, Kooner JS, Mooser V. (2008) Population-based genome-wide association studies reveal six loci influencing plasma levels of liver enzymes. Am J Hum Genet. 83, 520-528]**,** hence the link to Metabolic.

|  |
| --- |

**Supplementary Figure S2 Alignment of human 14-3-3 binding sites showing enrichment of phosphorylated residues in the -2 position relative to the phosphorylated 14-3-3-binding site**

The picture reports the alignment of the 245 manually-curated human 14-3-3 binding phosphorylation sites reported in the literature. The central serine or threonine responsible for 14-3-3 binding is highlighted in red. Where names are in yellow the corresponding sites are conserved in other members of the human 2R-ohnolog family and the central phosphosite aligns with a serine or threonine residue in the corresponding pro-ortholog from amphioxus and/or chiona. Post-translational modifications taken from PhosphoSite are highlighted according to the following colour code: 100 sequences contain a (S\T)Xp(S\T) motif (blue and green), of which 46 have the serine or threonine in the -2 position annotated as phosphorylated in PhosphoSite (blue). Other residues that can be phosphorylated are also indicated in blue, and other modified residues are indicated with the colour code indicated at the bottom of the figure. A Jalview [Waterhouse et al (2009) Jalview Version 2--a multiple sequence alignment editor and analysis workbench. *Bioinformatics* 25, 1189-1191] sequence consensus map is also shown.****

**Supplementary Figure S3A** **The extended phylogenetic tree of the vertebrate REEP1–4 and invertebrate REEP proteins**

A. As in the main Figure 3B, but with the individual genes in each vertebrate paralogy group shown. Blue branches show all of the REEP1 genes, red branches REEP2, green REEP3 and yellow REEP4. Bootstrap support values above 700 (out of 1000 replicates) are shown, except for some values close to the tips within the vertebrate paralogy groups for the sake of clarity, and the value uniting vertebrate REEP3 and 4, which is just less than 700. Figure S3B (next page) shows the region of the REEP proteins that was used for building the phylogenetic tree in this figure.

**Supplementary Figure S3B** **The extended phylogenetic tree of the vertebrate REEP1–4 and invertebrate REEP proteins**

B. The alignment of the REEP sequences was performed by MAFFT within Jalview (<http://www.jalview.org/>) and the purple highlighting reflects the percentage similarity across a position in the alignment. For the purposes of building the phylogenetic tree in Figure S3A, only the more conserved region of the REEP sequences up to position 186 in the alignment (following the motif LTLIR in human REEP2) was used.

| **** |
| --- |

**Supplementary Figure S4 Post-translational modifications surrounding the human 14-3-3 binding sites**

Of 245 manually curated 14-3-3 binding sites (Supplementary Table S1), 185 are annotated in PhosphoSite (www.phosphosite.org) as phosphorylated, and 3 as O-GlcNAc modified. The sequences (-8 / +8) surrounding the 185 sites were aligned and the post-transcriptional modifications in each column were counted (phosphorylation, acetylation, O-GlcNAc, sumoylation and ubiquitination retrieved from PhosphoSite). The same procedure was adopted with a collection of 185 phosphorylated sites randomly selected from PhosphoSite. The graph reports the number of post-translational modifications counted in each column of the alignment for the 14-3-3-binding sites (black) and random selection (gray). Similar results were obtained using 4 different random samples. The enrichment of modifications at -2 for the 14-3-3-binding sites is largely due to phosphorylations (see sequences in Supplementary Figure S4).

| **** |
| --- |

**Supplementary Figure S5 Biochemical regulation of human REEP proteins**

1. Effect of in vitro dephosphorylation on binding of REEP4 to 14-3-3s and Rab3GAP1, REEP4-GFP was isolated on GFP-Trap® agarose from lysates of transfected HEK293 cells, and then dephosphorylated *in vitro*, or not with λ phosphatase [as in Johnson et al (2011) Visualization and biochemical analyses of the emerging mammalian 14-3-3-phosphoproteome. *Mol Cell Proteomics* **10**:M110.005751]. These anti-GFP immunoprecipitates were immunoblotted with the indicated antibodies.
2. Interaction of REEP proteins with RAB3GAP1 and RAB3GAP2. HEK293 cells cultured in medium containing 10% (v/v) foetal bovine serum were transfected with plasmids to express the REEP proteins indicated. After 24 h, cells were serum-starved for 8 h (SS), then where indicated stimulated with phorbol-12-myristate-13-acetate (PMA, 100 ng/ml for 30 min). Proteins were isolated from cell lysates using GFP–Trap® agarose (Anti-GFP IP). Co-purification of endogenous RAB3GAP1, RAB3GAP2 and 14-3-3 was detected by immunoblotting with the indicated antibodies.
3. Interactions of a series of REEP 3/4 chimeric proteins with 14-3-3 and RAB3GAP1-RAB3GAP2. The protein segments were 1^st^ third (residues 1 to 80 of either REEP3 or REEP4, encompassing transmembrane hairpin), 2^nd^ third (residues 81 to 180 of either REEP3 or REEP4) and 3^rd^ third (residues 181 to 255 of REEP3, or 181 to 257 of REEP4). A 0 denotes that the indicated third was omitted. All proteins were GFP-tagged at the C-terminus. Proteins were isolated from lysates of transfected HEK293 cells using GFP-Trap® agarose, and the immunoprecipitates were immunoblotted with the indicated antibodies and probed for binding to digoxygenin-labelled 14-3-3 in a Far-Western assay. The lower panel shows blots of total cell extract.

**Supplementary table legends**

**Supplementary Table S1 The gold and silver standard human 14-3-3 binding proteins from the literature**

The table lists the UniProt accession number (UniProt_id), the UniProt name (UniProt_name), the 14-3-3 binding site position inside the protein (UniProt_residue), the amino acid type (Code), the peptide +6/-6 centred on the 14-3-3 binding site (Peptide), the PubMed accession number containing the literature evidence (PMID) of the proteins identified as 14-3-3 binding partners. The last column (Tag) contains the tag “GD” if the 14-3-3 binding site is identified (gold standard) or the tag “SL” if there is evidence of a phosphorylation-dependent 14-3-3 interaction without the identification of the binding site (silver).

**Supplementary Table S2 Ohnolog families containing one or more gold and silver standard human 14-3-3-binding proteins, and their disease associations**

For each protein identified in Supplementary Table S1, this table reports the Ohnolog family identifier (Family_id) and the number of proteins inside the family (Family_members). The Ohno_tag column contains the tag “Ohno” if the protein belongs to an ohnolog family or the tag “SINGLE” if the protein is present as a singleton in the human proteome. The Table also lists the UniProt accession number (UniProt_id), the UniProt name (UniProt_name), the associated disease (Disease), the disease class (Disease_class) for each protein, as defined in Supplementary Table S4. The last column (GD_tag) contains the tag “GD” if the 14-3-3 binding site is identified (gold) or the tag “SL” if there is an evidence of a phosphorylation-dependent 14-3-3 interaction without the identification of the binding site (silver). For the GD members the table reports the predicted sequence of the 14-3-3 binding sites (GD_sequence), if the site is conserved in *Branchiostoma* and/or *Ciona* (Conserved) and the presence of the S.S motif (Motif). The last column reports 14-3-3 binding sequences different from the lynchipin sites (Secondary_site). NB: There is no correspondence between the Family_id in Supplementary Tables S2 and S3.

**Supplementary Table S3 The high throughput (HTP) 14-3-3-affinity capture studies and ohnolog families**

For the articles under analysis (Experiment), the table lists the UniProt accession number (UniProt_id), the UniProt name (UniProt_name) the UniProt description (UniProt_description) and the Ohnolog family identifier (Family_id) of the proteins identified as 14-3-3 binding partners. The “Contaminant_degree” column reports how many times the protein has been identified in our list of 12 control experiments. The GD_tag column contains the tag “GD” if the 14-3-3 binding site is identified (gold) or the tag “SL” if there is an evidence of a phosphorylation-dependent 14-3-3 interaction without the identification of the binding site (silver). NB: There is no correspondence between the Family_id in Supplementary Tables S2 and S3.

**Supplementary Table S4 The associated diseases and phenotypes of the 14-3-3 ohnolog families**

The excel file contains a series of worksheets for the Developmental (class_developmental), Cancer (class_cancer), Immune (class_immune), Circulatory (class_cyrc), Metabolic (class_metabolic), Reproductive (class_reproduction), Cell proliferation (class_cell_proliferation) Neurological (class_neurological), Psychiatric (class_psych) and Other (other) disease type. These worksheets list the keywords and phrases used for computational assignment of proteins to each disease class as reported in Supplementary Table S2.
